# Importance of Hospital Infection Control

**DOI:** 10.7759/cureus.50931

**Published:** 2023-12-22

**Authors:** Dimple Kubde, Ankit K Badge, Sarita Ugemuge, Shivani Shahu

**Affiliations:** 1 School of Allied Health Sciences, Datta Meghe Medical College, Datta Meghe Institute of Higher Education and Research (DU), Nagpur, IND; 2 Department of Microbiology, Datta Meghe Medical College, Datta Meghe Institute of Higher Education and Research (DU), Nagpur, IND

**Keywords:** healthcare-acquired infection, healthcare workers, safety, prevention, practices, infection control

## Abstract

The increasing demand for healthcare-acquired infection (HAI) control practices and services has intensified the need to evaluate care quality. The World Health Organization (WHO) introduced an infection prevention and control (IPC) framework to mitigate the impact of HAIs, crucial for ensuring patient safety in hospitals. HAIs acquired after hospitalization pose significant challenges due to factors such as compromised immunity, invasive medical procedures, and antibiotic-resistant pathogens, which have dire consequences, including higher mortality rates and increased healthcare costs. Healthcare workers (HCWs) are critical in implementing IPC measures. Infection control programs that include strategies such as hand hygiene, personal protective equipment (PPE), environmental cleaning, and surveillance have become standard. However, challenges such as resistance to change, resource limitations, patient turnover, and variability in patient conditions persist. Strategies to maintain hospital infection control involve rigorous compliance monitoring, staff education, advanced technologies such as artificial intelligence (AI), machine learning (ML), telemedicine, and innovative sanitation methods. The future of hospital infection control may involve increased integration of environmental monitoring, antimicrobial stewardship, and patient participation while leveraging collaboration among healthcare facilities. The review highlights the criticality of hospital infection control and suggests trends and opportunities to strengthen prevention efforts and patient safety.

## Introduction and background

Healthcare-acquired infection (HAI) control practices and services are currently highly sought after due to increased demand. Consequently, there is a pressing necessity for implementing mechanisms to assess the quality of care delivered in this domain [1]. To promote a technical strategy and practical solutions for minimizing the damage caused by HAI, the World Health Organization (WHO) provided a framework for infection prevention and control (IPC). IPC is a key component of quality control and ensuring patient safety in hospitals. Preventing infections related to healthcare settings and reducing infection transmission are the main goals of IPC [2]. Direct or indirect contact with people and contact with contaminated materials are the two main ways infectious diseases are passed from one person to another [3]. Infection control programs have become standard practice in hospital care [4]. An infection contracted in a hospital or other healthcare facility, known as a HAI, is also called a nosocomial infection. Healthcare professionals, infection control experts, public health authorities, and patients are concerned about HAI [5]. All are affected by the transmission of infectious organisms in healthcare settings [6].

HAI is usually acquired after hospitalization and manifests 48 hours after admission to the hospital [7]. Factors such as compromised immunity in patients, invasive medical procedures, improper sanitation practices, and antibiotic-resistant pathogens significantly contribute to the prevalence of HAIs. The consequences are dire, leading to higher mortality rates, prolonged hospitalizations, increased healthcare costs, and a considerable burden on both individuals and healthcare organizations. Furthermore, these HAIs can be transmitted from patients to healthcare workers (HCWs) [8,9]. HCW plays an important role in the efficient implementation of IPC measures [10]. To reduce the adverse effects of HAI, hospitals should adopt hospital infection control practices [11]. The use of personal protective equipment (PPE) and good hand hygiene are essential elements for the prevention and control of HAI [11,12]. A crucial first step in creating an effective infection control program is determining the knowledge, attitudes, and practices that healthcare personnel currently have about infection control [13].

Inadequate funding for healthcare in general, the failure of facilities to implement efficient preventive measures, and inadequate training for HCW, particularly nursing staff, are just a few of the global health constraints that have an impact on infection prevention; a disproportionate burden is placed on least developed facilities [14]. Every HCW must practice infection control because it is one of their medical procedures [15]. In a variety of healthcare organizations, infection control procedures reduce the frequency of HAI, and as a result, they are now standard practice in the majority of medical centers in developed nations [16]. To make positive improvements in health, it is essential to recognize the dangers and restrictions of emerging infectious diseases and assess how they will affect current infection control practices [17]. The objective of this review is to highlight the importance of hospital infection control.

## Review

Methods

To conduct a comprehensive literature search, we used the following databases: MEDLINE and Google Scholar. For this review, we use the following search terms: ("Healthcare-acquired infection" OR "healthcare-acquired infection") AND ("Healthcare workers" OR "healthcare providers") AND ("Safety" OR "safety") AND ("Infection prevention" OR "infection prevention") AND ("Practices" OR "practices") AND ("Infection control" OR "infection control"). Articles published in the period 2001-2023 focusing on the importance of hospital infection control measures on reducing nosocomial infections, improving patient outcomes, enhancing healthcare quality, and ensuring patient safety were included in the current review. The article types include systematic reviews, case studies, and cross-sectional studies.

The IPC is essential to minimize the occurrence and spread of HAIs within healthcare settings. HAIs increase healthcare costs and strain available resources. Common factors contributing to HAI include many elements, including patient susceptibility due to compromised immunity, invasive procedures, extensive use leading to antimicrobial resistance, inadequate compliance with hand hygiene among HCWs, contaminated medical equipment, and suboptimal environmental hygiene [18]. Furthermore, lapses in proper sterilization techniques, overcrowding, and insufficient implementation of standardized infection control protocols collectively contribute to the proliferation of HAIs [19]. Infections caused by bacteria, viruses, and fungi are as follows (Table 1).

**Table 1:** Common hospital infections UTI: urinary tract infections; SSI: surgical site infections; BSI: bloodstream infections; GI: gastrointestinal; VAP: ventilator-associated pneumonia; CLABSI: central line-associated bloodstream infections; HAI: healthcare-acquired infection

Types of infections	Description
UTI	One of the most typical forms of HAI is UTI. They may occur if bacteria are inserted into the urinary tract by a catheter or other medical device [20].
SSI	SSI can affect the area around the incision as well as surrounding tissues and develop after surgery [12].
BSI	BSI occur when bacteria enter the bloodstream and can lead to life-threatening consequences such as sepsis [21].
GI	GI infections, which can cause dehydration and diarrhea, can be caused by bacteria or viruses that are spread by contaminated food, water, or surfaces [22].
VAP	VAP infections can harm the lungs and affect people who are using a ventilator or breathing apparatus [12].
CLABSI	CLABSI develop when bacteria enter the bloodstream through the central line [22].

The role of HCWs in preventing infections

Infection prevention is a key responsibility of HCWs, who also play a key role in patient education and ensuring that all elements of their nursing practice are supported by the latest scientific knowledge. Being supporters of patients, nurses are in a unique position to drive change and raise standards of patient care. To provide a secure environment for patients, nurses can use several tools. The single most important nursing action for infection prevention is hand washing, and it is a powerful tool in the nursing arsenal [23]. Nurses are required to wear PPE when handling body fluids. There are many other precautions that nurses can take to prevent infection at the bedside. They can provide a safe environment for patients in addition to practical bedside measures. This tactic helps the organization determine ways to improve the system and prevent future problems from occurring [24]. By applying their knowledge, skills, and judgment to carry out efficient and timely infection control activities, nurses can demonstrate leadership in preventing and managing the spread of infections in all roles and situations and maintain strict standards for patient safety [25,26].

Best practices and strategies for maintaining hospital infection control

Maintaining infection control in hospitals is essential to protect patients, medical personnel, and visitors. Here are some recommendations for best practices and tactics for managing hospital infection control [27].

Hand Hygiene

Hand hygiene is essential to prevent and limit the spread of infection [23]. It is probably the most obvious, auditable, and efficient IPC practice [28]. The thorough washing of the hands is one of the best methods to prevent the spread of infection. Healthcare personnel should wash their hands frequently [9]. Posters with images that promote the importance of hand hygiene should be placed near sinks and antiseptic items [29]. According to the WHO "Five Moments" model, HCWs should perform hand hygiene before and after touching a patient; before a clean or aseptic procedure; after the risk of exposure to body fluid; and after touching the surroundings or belongings of the patient [30]. The prevalence of HAIs can be significantly reduced if everyone practiced good hand hygiene [24].

PPE

To stop the spread of infectious organisms when caring for patients, healthcare personnel should wear proper PPE, such as gloves, gowns, masks, and eye protection [31]. PPE acts as a barrier between HCWs and potentially infectious materials [32]. Proper wear and disposal of PPE is essential to ensure safe practices. The effective use of PPE requires proper training, adherence to protocols, and regular evaluation of infection control practices [12,20].

Environmental Cleaning

Clean healthcare facilities appear pleasant, provide a sense of security, and improve patient satisfaction. To stop the transmission of diseases, hospital rooms, equipment, and surfaces need to be cleaned and disinfected regularly [33]. The use of appropriate disinfectants and cleaning agents helps to prevent cross-contamination [34]. By maintaining a clean and hygienic environment, hospitals can create a safer environment for patients, promoting their recovery and well-being [35].

Screening and Isolation

To prevent the infection from spreading to other patients, people who are colonized or infected with multidrug-resistant organisms (MDRO) should be detected by screening and isolation [36]. Hospitals typically have protocols in place to screen patients upon admission or arrival. Isolation precautions are used to separate patients with known or suspected infectious diseases from others to prevent the spread of the infection [37].

Education and Training

To keep healthcare personnel up-to-date with best practices, they should receive ongoing instruction and training on ways to prevent infections. This includes understanding the chain of infection, modes of transmission, and prevention strategies. Training should cover the implementation of standard precautions, which are the basic infection prevention measures used for all patients [38].

Sterilization and Disinfection

To prevent the transmission of infections, all medical equipment, especially reusable equipment, should be sterilized or disinfected before use. Sterilization is typically used for critical medical equipment that comes into contact with sterile body tissues. Disinfection refers to the process of reducing the number of microorganisms on surfaces, on instruments, or in the environment to a level that is considered safe [39].

Surveillance and Reporting

Infectious disease outbreaks should be detected and reported, and relevant control measures should be put in place in hospitals [9]. A functioning surveillance system is prospective, is comparative, and aims at achieving particular predetermined goals [40]. It accurately identifies the group at risk and forecasts how infection control efforts will turn out. Reporting is the process of transmitting surveillance data to the appropriate authorities or entities responsible for monitoring and regulating infection control practices [41].

Vaccination

To stop the transmission of disease, HCWs should get vaccinations against infectious diseases. Vaccination of HCW is essential to protect their health and prevent them from contracting and spreading infections [12]. HCWs should follow local guidelines and stay up-to-date with vaccination recommendations provided by their employers and public health authorities to ensure effective infection control within the hospital setting [41]. By implementing these best practices and strategies, infectious disease transmission can be prevented and maintained in hospitals [42].

Technology and innovation in hospital infection control

The improvement of hospital infection control has been greatly aided by technology and innovation. Here are some examples of how technology and innovation are being used in hospital infection control:

Ultraviolet (UV) Disinfection Systems

UV light killed bacteria and viruses by UV radiation. UV disinfection systems are being used in hospitals to disinfect patient rooms, operating rooms, and other areas where infectious organisms may be present. These systems use UV lamps to reduce the risk of HAI [8].

Electronic Hand Hygiene Monitoring

One of the best strategies to stop the spread of infection in hospitals is to practice good hand hygiene. Electronic hand hygiene monitoring systems use sensors to track when healthcare personnel enter and exit patient rooms and can track whether they wash their hands or apply hand sanitizer. This technology can help hospitals identify areas where hand hygiene compliance is low and improve overall compliance rates [40].

Antimicrobial Surfaces

In hospitals, bed rails and door knobs are two frequently touched items and can host bacteria and viruses. Antimicrobial surfaces are created to eliminate viruses and bacteria immediately upon touch, reducing the chance of transmission. Copper or silver, which have antibacterial qualities, can be used to create these surfaces [26].

Advanced Air Filtration Systems

In healthcare facilities, particularly in areas such as operating rooms and intensive care units, infections can spread through the air. Modern air filtration systems are capable of removing germs and viruses, as well as other airborne particles. The risk of spreading an infection through the air can be reduced with the use of these systems [40].

Electronic Patient Monitoring

Using electronic patient monitoring systems to track vital signs and other medical data, healthcare professionals can identify patients who could be at risk of infection. When a patient's condition changes, these systems can notify appropriate healthcare professionals, allowing earlier intervention and possibly reducing the risk of infection [6]. Technology and innovation are becoming more crucial to hospital infection control, reducing the incidence of HAIs, and improving patient outcomes [40].

Challenges in implementing effective hospital infection control

HCWs often resist adopting new infection control protocols due to their familiarity with existing practices. The shift to new methods can be met with reluctance or hesitancy, stemming from the comfort and habituation to established procedures. Embracing change in healthcare settings can be challenging and requires extensive training and support mechanisms. Providing detailed education, demonstrations, and ongoing guidance is crucial to facilitate a smooth transition and ensure the effective implementation of new infection control measures. Supportive leadership and a culture that encourages adaptation and continuous improvement are vital to overcome this resistance. Effective infection control is highly dependent on having adequate resources. These encompass a range of necessities, such as sufficient PPE, access to high-quality cleaning materials, financial support for maintenance and procurement, and a well-educated workforce. Without these essential resources, the implementation and sustainability of proper infection control become significantly compromised. Shortages or inadequacies in these resources not only affect patient care but also put HCWs at risk, potentially leading to increased infection transmission within healthcare settings [5]. Healthcare facilities, particularly hospitals, operate in an environment characterized by a constant influx of people, resulting in a dynamic and bustling atmosphere. This high turnover poses a continuous challenge to maintain hygiene and cleanliness and hygiene standards. The sheer volume of people entering and exiting increases the risk of cross-contamination and the spread of infections. Ensuring rigorous cleaning protocols, adequate isolation measures, and strict adherence to infection control practices become crucial in managing this constant flow of patients and visitors [43]. Patients in healthcare settings have a variety of medical conditions, which require customized infection control strategies. A singular and standardized approach may not effectively address the varied needs of patients with different medical problems. Tailoring infection control measures to specific conditions becomes challenging, as it requires a nuanced understanding of various diseases, their transmission modes, and appropriate preventive measures. Implementing a flexible approach that can accommodate this variability is essential for complete infection control within healthcare facilities [34]. Effective infection control is highly dependent on seamless communication and collaboration among different stakeholders within healthcare settings. In larger hospitals or institutions with numerous departments and a wide array of personnel, maintaining clear communication channels and fostering collaboration can be challenging. This can lead to gaps in conveying crucial information related to infection control protocols, resulting in inconsistent practices or misunderstandings between staff, patients, and their families. Establishing robust communication strategies and encouraging interdisciplinary collaboration are crucial to bridge these gaps and ensure cohesive infection control efforts [38]. The proficiency in infection prevention methods is crucial to successful infection control. However, inadequate or insufficient training in these methods can hinder the implementation of effective infection control measures. Without proper education and ongoing training programs, healthcare personnel may lack the skills and knowledge to implement preventive measures accurately. Investing in comprehensive training initiatives and continuous education programs is crucial to empower HCWs with the expertise needed to combat infections effectively [43]. Patient adherence to infection control measures, such as proper hand hygiene or adherence to isolation protocols, is crucial to prevent the spread of infections within healthcare facilities. However, noncompliance with these measures poses a significant risk. Despite efforts of healthcare care providers to educate and encourage patients to follow infection control guidelines, individual behaviors and attitudes toward these practices can vary. Patient education, clear communication of the importance of these measures, and the creation of a supportive environment for compliance are essential strategies to mitigate the risk associated with patient behavior in infection spread within healthcare settings [38]. The challenges in implementing effective hospital infection control are as follows (Table 2).

**Table 2:** Challenges in implementing effective hospital infection control PPE: personal protective equipment; HCWs: healthcare workers

Challenges	Description
Resistance to change	HCWs may be resistant to change and new protocols. They may be used to certain practices and find it difficult to adopt new infection control measures [5].
Lack of resources	Adequate resources, such as PPE, cleaning materials, finance, and educated employees, are all required for effective infection control. In the absence of these resources, implementing and maintaining effective infection control may be difficult [5].
High patient turnover	Hospitals are busy places with lots of people entering and exiting. Due to this, keeping a hygienic and clean workplace may be challenging [43].
Variability in patient conditions	Patients may need various infection control strategies according to their various medical problems. Implementing a common infection control technique may be difficult due to this variation [34].
Limited communication and collaboration	Effective communication and collaboration among medical staff, patients, and patient's relatives are essential for effective infection control. This could be challenging in a hospital setting with several departments and personnel [38].
Inadequate training	Healthcare personnel may not have sufficient training methods for preventing infections. They may not be able to execute effective steps to prevent infections without the right training [43].
Patient behavior	Patient noncompliance with infection control measures, such as hand hygiene and isolation measures, might increase the possibility of an infection spreading [38].

Compliance and monitoring

Hospital infection control measures, which are designed to stop the spread of contagious diseases among patients, healthcare providers, and visitors, must be followed with compliance and monitoring to be effective. The following are some essential actions that hospitals can take to ensure compliance and efficient monitoring. Hospitals should create rules and procedures that outline exactly what actions HCWs must take to stop and manage infections. These regulations must be updated and reviewed frequently and must be founded on current evidence-based recommendations [31]. Training of all hospital personnel on infection prevention techniques is necessary. Continuous training, many training sessions, reminders, and compliance feedback are required as part of this instruction [43]. Hospitals should develop methods for monitoring and feedback to ensure that staff adhere to infection control procedures. This can mean keeping an eye on workers, keeping an eye on infections, and giving them feedback on their compliance [44]. Hospitals should constantly review their infection control protocols to identify areas for improvement. This can involve performing audits, evaluating surveillance information, and collecting feedback from employees [7]. Open communication with patients, visitors, and healthcare professionals can help identify areas for improvement and encourage adherence to infection control protocols [45]. By efficiently implementing and monitoring their infection control measures, hospitals can help prevent the spread of infectious diseases and protect the health of patients, healthcare providers, and visitors [44].

The future of hospital infection control

Hospitals are leveraging artificial intelligence (AI) and machine learning (ML) to proactively identify and curb the spread of infections. These technologies can detect patterns and predict potential outbreaks. AI-powered systems monitor staff compliance with hand hygiene regulations and identify infections on surfaces, alerting staff for timely intervention [26]. Telemedicine has emerged as a vital tool in infection control, enabling remote consultations and monitoring. Healthcare professionals can receive training and education on infection prevention techniques remotely, reducing the risk of transmission within healthcare settings [35]. Hospitals are exploring advanced sanitation methods such as UV light, electrostatic sprayers, and hydrogen peroxide vapor to efficiently clean surfaces and equipment. These technologies not only improve cleaning effectiveness but also significantly reduce the need for extensive manpower, thereby optimizing costs [44]. Environmental monitoring systems provide real-time data on factors such as temperature, humidity, and air quality that affect infection control. Hospitals use this information to identify potential sources of infection and take proactive measures [45]. Antibiotic stewardship programs are crucial in preventing infection-resistant infections. Hospitals are developing comprehensive antimicrobial management initiatives to optimize antibiotic use, thus mitigating the risk of antibiotic-resistant infections [32,34]. Hospitals are integrating infection control into broader patient safety programs, recognizing its crucial role in overall patient safety. Prioritizing infection prevention, early detection, and rapid response to outbreaks within these programs is essential for effective control [32]. Hospitals are fostering increased collaboration to combat infection spread, acknowledging that infection control is a shared responsibility. This involves sharing best practices, information, and resources among healthcare facilities. Formalized collaborations and networks are likely to promote infection prevention in the future [4]. Recognizing the importance of patient involvement in infection control, hospitals are educating and engaging patients in their care. Empowering patients with knowledge about infection prevention methods contributes significantly to reducing HAIs [42]. Possible trends and opportunities may influence hospital infection control in the future (Table 3).

**Table 3:** Emerging trends and opportunities for hospital infection control AI: artificial intelligence; ML: machine learning; UV: ultraviolet

Trends and opportunities	Description
Use of AI and ML	Hospitals may take proactive steps to stop the spread of infection with the use of AI and ML, which can assist spot patterns and forecast outbreaks. AI-powered methods, for instance, can track staff compliance with hand sanitation regulations or identify infections on surfaces and notify staff to take appropriate action [26].
Implementation of telemedicine	Through the use of remote consultations and monitoring, telemedicine can lower the risk of infection transmission. Healthcare professionals can be trained and educated remotely on infection prevention techniques through telemedicine [35].
Advanced disinfection technologies	Hospitals are experimenting with advanced sanitation techniques like UV light, electrostatic sprayers, and hydrogen peroxide vapor to clean surfaces and equipment. In addition to being more efficient than conventional cleaning techniques, these technologies can significantly reduce manpower costs [44].
Integration of environmental monitoring	Environmental monitoring systems can offer real-time data on temperature, humidity, air quality, and other factors that could affect the control of infections. Using this information, hospitals can identify and treat possible infection sources [45].
Increased focus on antimicrobial stewardship	Antibiotic stewardship programs are crucial for preventing the spread of infections that are resistant to antibiotics. Hospitals are creating comprehensive antimicrobial stewardship programs to optimize antibiotic usage and reduce the risk of antibiotic-resistant infections [32,34].
Integration of infection control into patient safety programs	Hospitals are beginning to incorporate infection control into more extensive patient safety programs since it is an essential part of patient safety. Focusing on infection prevention, early infection detection, and quick outbreak response is necessary to achieve this. By integrating it into patient safety programs, hospitals can make infection control a top priority [32].
Increased collaboration between healthcare facilities	Hospitals are collaborating more to stop the spread of infections as infection control is a shared responsibility. This involves exchanging best practices, information, and resources to enhance infection control among healthcare facilities. To promote infection prevention, hospital networks and collaborations may become more formalized in the future [4].
Patient involvement	Patients play an important role in hospital infection control. The risk of infections related to healthcare can be decreased by involving patients in their care and educating them about infection prevention methods [42].

## Conclusions

Hospital infection control is essential to protect patients, HCWs, and the general community from HAI. The intricate web of challenges, from high patient turnover to varying patient conditions, underscores the need for robust strategies. These strategies include best practices such as strict hand hygiene, PPE, environmental cleaning, surveillance, and innovative technologies such as AI and UV disinfection systems. Overcoming challenges requires resource allocation, effective communication, ongoing training, and patient participation. The future of infection control lies in the embrace of advanced technologies, the integration of infection control into patient safety initiatives, the promotion of collaboration, and the empowerment of patients. Ultimately, by actively involving patients in their own care and promoting education and awareness, a culture of infection prevention can be promoted, leading to a safer healthcare environment for all. As hospitals evolve, these trends and opportunities will shape the landscape of infection control, emphasizing the proactive pursuit of patient safety and well-being.
